# Healthcare Provider Advocacy for Primary Health Care Strengthening: A Call for Action

**DOI:** 10.1177/21501319221078379

**Published:** 2022-03-15

**Authors:** Flora Kuehne, Laura Kalkman, Shiv Joshi, Wunna Tun, Nishwa Azeem, Dabota Yvonne Buowari, Chioma Amugo, Per Kallestrup, Christian Kraef

**Affiliations:** 1 LMU University Hospital, Institute for General Practice and Family Medicine, Munich, Germany; 2 Medische Centrum Leeuwarden, Leeuwarden, The Netherlands; 3 Mahatma Gandhi Institute of Medical Sciences, Sewagram, India; 4 Independent Researcher, Yangon, Myanmar; 5 Independent Researcher, Lahore, Pakistan; 6 University of Port Harcourt Teaching Hospital, Port Harcourt, Nigeria; 7 Ashford and Saint Peter’s Hospitals NHS Trust, Chertsey, UK; 8 Aarhus University, Aarhus, Denmark; 9 Rigshospitalet, University of Copenhagen, Denmark

**Keywords:** primary health care, advocacy, health care provider, health systems strengthening

## Abstract

Primary Health Care (PHC) is the backbone of health systems and a cornerstone of Universal Health Coverage. In 2018, political commitment to PHC, including a comprehensive approach based on essential care throughout the lifespan, integrated public health functions, and community empowerment was reaffirmed by international stakeholders in Astana. As recent events exposed weaknesses of health care systems worldwide, growing attention has been paid to strengthening PHC. While the role of care providers as health advocates has been recognized, they may lack skills, opportunities, and resources to actively engage in advocacy. Particularly for PHC providers, guidance and tools on how to advocate to strengthen PHC are scarce. In this article, we review priority policy areas for PHC strengthening with relevance for several settings and health care systems and propose approaches to empower PHC providers—physician, non-physician, or informal PHC providers—to advocate for strengthening PHC in their countries by individual or collective action. We provide initial ideas for a stepwise advocacy strategy and recommendations for practical advocacy activities. Our aim is to initiate further discussion on how to strengthen health care provider driven advocacy for PHC and to encourage advocates in the field to reflect on their opportunities for local, national, and global action.

## Introduction

Primary Health Care (PHC) has been defined as *“a whole-of-society approach to health that aims at ensuring the highest possible level of health and well-being and their equitable distribution by focusing* *on people’s* *needs as early as possible along the continuum from health promotion and disease prevention to treatment, rehabilitation and palliative care, and as close as feasible to* people’s *everyday* environment.”^1^ PHC can address the majority of health needs of patients throughout their life course. However, globally only about half of the population has full coverage with essential health services, and access to health care services and quality of care varies widely.^2,3^

Political commitment to PHC was reiterated 40 years after the *Alma-Ata Declaration* by international stakeholders, national health ministers and delegates, health professionals, civil society, and academia at the Global Conference of Primary Health Care 2018 endorsing the *Astana Declaration*.^2^ Based on the core principles of Alma-Ata,^4^ including equity, social justice, universal access to care, community participation, and intersectoral collaboration, the PHC approach enshrined in the Astana Declaration comprises comprehensive, integrated care for the individual throughout the lifespan. It addresses the broader determinants of health through public policies and cross-sectoral action and empowers individuals, families, and communities to take an active role in health.^2^ In addition, the renewed declaration embraces the concepts and efforts toward universal health coverage (UHC) and the sustainable development goals (SDGs).^5-7^

Over the last decades, a growing body of evidence has demonstrated the capability of strong PHC systems to contribute to resilient and cost-effective health systems and has thereby underlined its importance as the programmatic engine of UHC.^6^ However, a lack of political support for primary care services and global funding priorities for vertical health programs and highly specialized care (secondary or tertiary) has resulted in weak PHC services in many countries.^7-9^ Worldwide out of pocket spending for PHC is still concerningly high at 59% of total PHC expenditure worldwide.^10^ Also, the shortage of around 18 million health care workers continues to impede the availability of health care services, affecting low and middle income countries disproportionally. In 2013 there was a staffing gap of 2.6 million doctors, 9 million nurses, and 5.9 million other health care workers required to achieve the SDG health targets.^11^ Especially in PHC, low numbers of physicians and nurses compared to hospital-based specialists have been reported in many countries.^12^ For instance, the share of general practitioners among all physicians has been decreasing, with only 29% in OECD countries in 2016 and an accentuated shortage of PHC physicians in remote areas.^13^ Also, inappropriate task distribution within PHC teams and competency gaps of health care workers represent barriers to effective and high-quality services.^14^

Several strategies have been identified to address the challenges that PHC faces worldwide, but documentation and reflection on the role and the “how” of health care provider involvement in local, national, and global advocacy for strengthening PHC is lacking.

## PHC Providers as Advocates for PHC

With the term PHC providers we refer to all occupations engaged in organizing and delivering PHC, including unpaid caregivers, volunteers, and informal health workers. Depending on the respective setting this can comprise general practitioners or family medicine specialists, nurses, auxiliary nurses, pharmacists and pharmacist assistants, public health nurses, community health workers, or social workers. In a broader sense, also professionals responsible for the strategic functioning and planning of PHC such as researchers, epidemiologists, policy makers, managers, or educators are part of the PHC workforce.^15^

To strive toward the principles stated in the Astana Declaration and to realize the vision of strong and comprehensive PHC, strong leadership and advocacy on the macro- (health system), meso- (health care organization, community), and micro-level (PHC team, health care facility) are needed.

Being a health advocate has been described among the core competencies of a physician and is increasingly being considered in curricula of medical schools.^16-20^ Similar movements have been observed for community health workers,^21,22^ nurses,^23-25^ and pharmacists^26,27^ postulating advocacy as a critical responsibility for health care providers and the need for respective training.

Educational strategies should be developed for different professions and integrated into the respective curricula and extracurricular learning opportunities. Establishing health advocacy frameworks and definitions of advocacy core competencies has been proposed as a basis for design and adaptation of health advocacy training.^19,26,28,29^ Educational contents can comprise health policy and legislative advocacy, communication skills, grassroot advocacy, translating research for wider audiences, community partnership, and teaching advocacy to other professions.^19,26,30^ Regarding educational methods, the importance of longitudinal and particularly hands-on training opportunities have been emphasized such as community placements, implementing individual or group health advocacy projects, writing or lobbying to legislators or the press, or simulating international health governance processes.^19,28,31-33^

There is no one common definition for advocacy by health care providers. In the present article we refer to advocacy as “Action by a physician to promote those social, economic, educational, and political changes that ameliorate the suffering and threats to human health and well-being that he or she identifies through his or her professional work and expertise” as defined by Earnest et al.^28^ Further, we extend this definition to all PHC providers (not only physicians) mentioned above. Apart from individual action, health care providers many times participate in professional organizations or associations. This serves to unify normative beliefs and policy interests, to increase public or political awareness and unite actions. Such organizations have been described to often focus on certain stages of the policy process, particularly on the agenda-setting of policies and the policy implementation.^34^

Further, health care providers are often involved in clinical research, either affiliated with academic, governmental, or private institutions. Apart from individual or collective action as providers, as researchers their role in the policy process extends to provide policy makers with evidence-based information or recommendations on the topic related to a certain policy.^35^

Physicians are in a powerful position to engage in advocacy, as they usually enjoy high confidence by the public and have the academic knowledge and understanding of the medical aspects and interdependencies with broader determinants of health.^28^ Physicians act as service providers, but moreover as leaders of health care teams, innovators of new approaches to provide health care services, and as researchers.

Advocacy and leadership by all non-physician PHC providers are equally important. Advantages of different health professions within this group include for example an increased direct patient contact, a trustful “eye-level” patient-provider relationship and a deep understanding of patients’ environment and social situation through home visits, community action, or belonging to same or similar marginalized groups (shared vulnerability).^22,25-27^ On the other hand, particular challenges to advocacy involvement are posed to these professional groups for example through lack of opportunities to advocate in work-hours, restrictions in their scope of care, hesitancy to act politically, lack of integration within the medical workforce, and perceived disaffirmation as advocates within the broader healthcare workforce.^22^

All PHC providers, based on their duty to meet the health needs of their patients, do not only act as health advocates for their individual patients, but can address the “upstream-factors” of communities’ and populations’ health through public policy.^16^

Their perspective, therefore, is crucial in developing, implementing, and evaluating policies to measure, improve, expand, or restructure health services.

## Policy Options for PHC

Interventions and best practice examples to strengthen PHC and health systems have been reviewed before, based on the best available evidence to inform political choices.^36,37^ Policy options often remain generic as they have to be adapted to the respective setting before implementation and best practice examples in many cases do not explicitly elaborate on the PHC provider advocacy efforts required or desired. Apart from that, research about policy options is predominantly focused on recommendations for political decision-makers (governments and ministries). Underlining the importance of PHC providers and academia in public health policy, this article seeks to propose policy recommendations that are relevant for care providers, initiate further discussion on how to strengthen health care provider driven advocacy for PHC and to encourage those active in the field to reflect on their unique opportunities for local, national, and global action.

In Table 1, we present several policy options to strengthen PHC to propose possible fields of action.

**Table 1.** Advocacy Options for PHC. *Oriented on.*^3,36,37^

System	
Governance and leadership	• Lead the development of a shared vision for PHC within the respective health system, local government, or community • Ensure acceptability of PHC policies for providers • Enforce accountability of policies (e.g. adapted to population needs) • Contribute to building evidence to inform policy decisions Advocate for a health-in-all-policies approach
Health financing	• Prioritize PHC within the health system and optimize resource use within PHC services • Tailor packages of guaranteed PHC services to the needs of the local population • Ensure minimal out-of-pocket spending for patients • Identify, analyze and promote innovative payment schemes that are fair and encourage services that improve health outcomes and control for special needs of vulnerable patient groups and socioeconomic disparities Align the compensation of PHC providers and providers of specialized health services
Adjustment to population health needs	• Ensure social accountability and feasibility of prioritized health service decisions by engaging in local priority setting • Collaborate with community representatives and support Community Health Action Plans • Develop surveillance systems and support relevant and complete data generation and collection • Get actively involved in the development of relevant research questions and methodologies within the local, regional, or national context • Safeguard the use of population health data to improve service delivery Lead and promote innovation and learning within the health system
Inputs	
Drugs and supplies	• Enforce a sound supply chain management including the prioritization of PHC facilities in case of emergencies • Ensure the assessment of quality, safety and performance of health products Collaboration of clinicians and pharmaceutical professionals to improve local supply chain monitoring, pharmacovigilance and, patient safety, as well as analyze prescription behaviors in terms of quality management and learning and to innovate information systems
Facility infrastructure	• Make the case for increased investments in infrastructure of PHC facilities, equipment, and safety precautions where most needed Advocate for more accessible health facilities taking into account the needs and barriers for the population
Information systems	• Establish functional information systems and ensure integration into clinical practice and usefulness for providers • Promote interoperability and interconnectedness of information systems to improve coordination of care Emphasize the need for feedback loops to ensure that analysis and measurement of generated health data serves to improve care and learning of health systems
Workforce	• Call for high quality and context-specific training • Ensure the alignment of educational strategies with health service needs of the population • Extend academic systems and networks to bring medical education and supervision to PHC centers and rural areas Contribute to effective recruitment schemes to attract workforce to rural settings
Funds	• Promote transparency and fairness in the assignment of funds • Ensure cost-effectiveness with a focus on health outcomes rather than profitability • Increase provider autonomy to manage funds at facility or community level to enable flexibility and responsiveness of health services Enforce trustworthy record-keeping and combat informal payments and bribes
Service delivery	
Population health management	• Promote a bottom-up approach based on population needs • Support community involvement, for example, through Community Health Management Committees • Integrate primary care functions with public health functions and collaborate in multisectoral partnerships on micro-, meso-, and macro-level Assess local needs by collecting individual and population level data and strengthen partnerships between local health facilities and local government
Facility organization and management	• Increase multidisciplinary of PHC teams for better skills matching, provider satisfaction, and availability of services • Improve coordination of providers, case management, and patient flow • Enforce robust and reliable referral mechanisms • Partner with hospitals and community care to enhance integration of care Implement quality management mechanisms at all levels of care to provide effective, reliable and high-quality processes, and to eradicate malfunctions
Access	• Broaden the use of electronic communication and digital technology • Safeguard clinical appropriateness and sound working conditions while extending office hours or sites • Extend mobile health care facilities Address special needs and barriers of patients to access PHC services
Availability of effective PHC services	• Amend task shifting to non-physician health care professionals and community health workers while ensuring that PHC teams are led by physicians • Enforce decent working conditions and remuneration • Support professional development of PHC providers by implementing training courses designed to the local context • Enhance the recognition of PHC physicians within medical specialties, levels of care and research Embrace implementation of effective and safe digital health services (e.g. smart-phone based applications) to increase access and adherence to health care
High-Quality PHC	• Establish high quality standards including treatment guidelines, checklists, decision support tools, and quality management mechanisms that are specific for the primary care setting • Promote performance-based accountability systems • Contribute to research and measuring quality of care Ensure high quality of integrated services by partnering with secondary care specialists and hospitals

Policy options were selected from a comprehensive review of publications by the WHO^36^ and the Primary Health Care Performance Initiative (PHCPI),^37^ a partnership of international organizations, private institutions, and philanthropies. Whereas the former is a collection of evidence-informed improvement options and country case studies, the latter is a synthesis of literature review, regional reports, country case studies, and expertise by the International Advisory Group on Primary Health Care. Selection of policy options was made by a group of young physicians working in the field of PHC in Europe, Asia, and Africa according to perceived relevance for PHC providers based on their personal experience.

The policy options were categorized by using the Primary Health Care Performance Initiative (PHCPI) conceptual framework^38^ assigning them to the domains system, inputs and service delivery, and its subdomains. The PHCPI conceptual framework describes components of high functioning PHC systems and was chosen due to its special focus on service delivery aspects, thereby differing from many other existing frameworks rather emphasizing inputs and outputs.^38^The final selection of policy options and the assignment to the respective domains of the framework was discussed and confirmed by consensus of the authors (practitioners from Denmark, Germany, India, Myanmar, The Netherlands, Nigeria, and Pakistan).

The advocacy options presented in Table 1 should serve as an overview and suggestion for PHC providers and can help select personal or organizational advocacy priorities. PHC advocates might choose a field that has been identified as urgent from the personal practice, that has been raised by patients or colleagues or that has been published by academic or governmental institutions, for example originating in a burden of disease or economic burden.

## Ways to Engage in Advocacy

There is currently a lack of systematic guidance for PHC providers on how to engage in advocacy to strengthen PHC at different levels. In Table 2 we collated stepwise recommendations on how to set advocacy priorities, plan advocacy, implement, and evaluate advocacy.

**Table 2.** Advocacy Step by Step. *Oriented on*.^39-43^

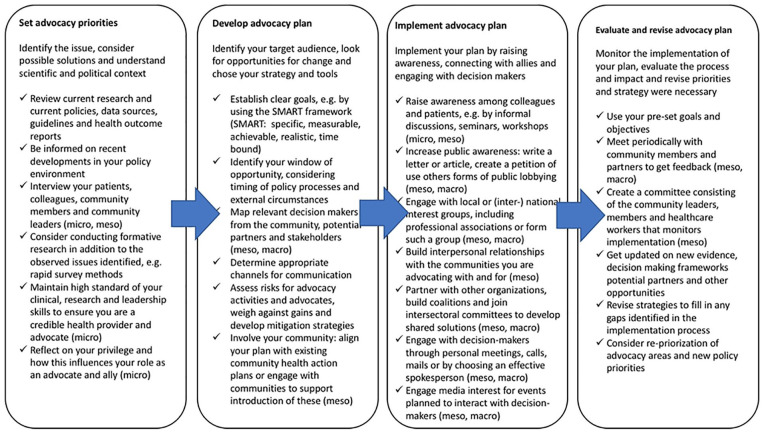

When setting advocacy priorities, it is important to have a clear overview of relevant evidence and to explore whether there are research gaps that need to be addressed. Engagement with patients, communities, and colleagues to understand and gain their perspectives and concerns on the issue is advised. This will help determine appropriate policy changes to advocate for based on criteria such as burden of the health issue in your community, effectiveness of interventions, ethical values behind the change, costs, and acceptability of the intervention.^44,45^

While further developing an advocacy plan, creating an overview of the decision-making system and decision-makers’ motivations and interests will help focus advocacy efforts and possible collaborations. Risks of advocacy, for example political or cultural offense against advocates and health care workers, strain on existing relationships, and impact on organization’s neutrality or reputation should be assessed for advocacy activities, messages, and messengers. While particularly considering risks for the most vulnerable involved, mitigation strategies are to be developed.^42^

Taking into account the historic context of health policy decisions as well as (international) epidemiological, natural or political events and developments help the identification of an appropriate timing for advocacy efforts.

Apart from engaging directly with decision-makers, it is alternatively possible to drive change by influencing a secondary audience, such as the community, that has access to decision-makers.^46^

Which stakeholders or decision-makers to target further depends on the organizational structure of the local practice, health care organization or health system. There are no one-size-fits-all recommendations. For instance in countries with centralized (national) health systems advocacy strategies related to the categories *governance and leadership* and *health financing*, but also some advocacy strategies related to *funds* and *High-Quality PHC* might be addressed at the macro level, targeting for example the national government, ministries of health or accreditation authorities. In federal or regionally organized health systems, the respective bodies at regional level might be rather addressed. Some advocacy strategies from the categories *adjustment to population health needs*, *population health management*, and *access* on the other hand might necessarily require involvement of regional health authorities, local research institutions, or the communities and therefore be rather suited for meso level action in many settings. Finally, some advocacy strategies from the categories *facility organization* *and management*, *high-quality PHC* or *access* will be able to be addressed at the micro level in many settings, starting with action in the advocate’s own work place or organization. Aspirations for a broader reach or similar standards beyond a certain health facility or local institution, however, will require action on the meso or macro level as well. In addition, the level of advocacy action depends on the capacity and aspirations of the advocate. For instance, it might be easier to change something in local practice or within the community (micro, meso) due to the close contact with (few) stakeholders involved.

When implementing the advocacy plan there are a diversity of ways to influence decision-makers directly or indirectly and to raise awareness among the public. If advocating in a team or association it will be useful to have a common policy document stating the position, claims, arguments, evidence, and clearly distributed responsibilities. Designating contact persons for each stakeholder addressed can help to create stronger personal trust-based relations.

Regarding the evaluation of the advocacy plan and efforts, considering the perspectives of persons outside your practice, organization, or community is crucial. When analyzing progress based on set objectives, barriers, and facilitators for implementation should be identified. This will help to make the advocacy plan more effective. Recognizing failures and reflecting on ways to prevent or improve in the future can contribute to a no-blame culture and personal and organizational learning. Apart from the internal factors discussed above, it is important to consider external factors such as updated evidence, changes in the political environment or new windows of opportunities on an ongoing basis, which potentially require a change or adaptation of advocacy priorities.

## Conclusion

Strong PHC systems bear a tremendous potential and are advocated for various reasons. It is reckoned as the strategy to improve population’s health and wellbeing and as a prerequisite to achieve UHC, contributing to various issues including equity matters, cost-effectiveness of health services and resilience of health systems.^1,5^ While international attention on PHC has been reiterated in Astana some years ago, the recent COVID-19 pandemic with its challenges on health systems and health care service delivery produced momentum for investments as well as policies in health care and, advocacy-wise, can be seen as a window of opportunity.

PHC providers play an important role in strengthening PHC through leadership in health care practice and by engaging in advocacy. Their clinical experience, medical knowledge, and position within PHC teams, health systems and communities make PHC providers particularly valuable and influential in political processes to improve health and shape health services and systems.

There is a lack of concrete recommendations on health care provider driven advocacy for PHC. This is particularly the case for non-physician and non-administrative health care workers, who we propose should be actively encouraged and included in PHC related advocacy strengthening. Further research is needed examining the involvement of PHC providers in policy processes and their influential power in certain stages of the policy process. Special attention should be paid to differences between different professions within the group of PHC providers and respective chances and barriers. Apart from that, evidence on the applicability and effectiveness of policies and implementation strategies particular to the PHC setting would be important for health care providers in order to engage in advocacy. To develop this field the authors call for increased sharing of best practices on provider advocacy to strengthen PHC and their incorporation into educational strategies and curricula for care providers. To stimulate further discussion, reflection, sharing of best practice and research on this topic we offer an initial overview of potential priority areas for advocacy on PHC strengthening, a proposal for a stepwise approach to developing advocacy initiatives and recommendations for their local, national, or global implementation.

With this article, the authors hope to encourage those active in the field to reflect on their unique opportunities for local, national, and global action and call on PHC providers to become active advocates and contribute to strengthen PHC.
